# Trichostatin A Stabilizes the Expression of Pluripotent Genes in Human Mesenchymal Stem Cells during *Ex Vivo* Expansion

**DOI:** 10.1371/journal.pone.0081781

**Published:** 2013-11-27

**Authors:** Bing Han, Jing Li, Zhilong Li, Ling Guo, Shan Wang, Peishu Liu, Yaojiong Wu

**Affiliations:** 1 Department of Obstetrics and Gynecology, Qilu Hospital, Shandong University, Shandong, China; 2 The Shenzhen Key Laboratory of Health Sciences and Technology, Graduate School at Shenzhen, Tsinghua University, Shenzhen, China; 3 School of Life Sciences, Tsinghua University, Beijing, China; Rutgers - New Jersey Medical School, United States of America

## Abstract

**Background:**

Mesenchymal stem cells (MSCs) have been considered as ideal cells for the treatment of a variety of diseases. However, aging and spontaneous differentiation of MSCs during culture expansion dampen their effectiveness. Previous studies suggest that *ex* *vivo* aging of MSCs is largely caused by epigenetic changes particularly a decline of histone H3 acetylation levels in promoter regions of pluripotent genes due to inappropriate growth environment.

**Methodology/Principal Findings:**

In this study, we examined whether histone deacetylase inhibitor trichostatin A (TSA) could suppress the histone H3 deacetylation thus maintaining the primitive property of MSCs. We found that in regular adherent culture, human MSCs became flatter and larger upon successive passaging, while the expression of pluripotent genes such as Oct4, Sox2, Nanog, Rex-1, CD133 and TERT decreased markedly. Administration of low concentrations of TSA in culture significantly suppressed the morphological changes in MSCs otherwise occurred during culture expansion, increased their proliferation while retaining their cell contact growth inhibition property and multipotent differentiation ability. Moreover, TSA stabilized the expression of the above pluripotent genes and histone H3 acetylation levels in K9 and K14 in promoter regions of Oct4, Sox2 and TERT.

**Conclusions/Significance:**

Our results suggest that TSA may serve as an effective culture additive to maintain the primitive feature of MSCs during culture expansion.

## Introduction

Mesenchymal stem cells (MSCs) are multipotent stem cells [1,2]. Accumulating evidence suggests that MSCs have profound therapeutic potential for a variety of diseases such as myocardial infarction, neural diseases and wound healing [3,4]. Due to encouraging preclinical results, a large number of clinical trials for various diseases are underway [5]. 

MSCs are distributed in a variety of tissues such as the bone marrow and adipose tissue [1,2,6], but represent a rare cell population in tissues. For example, MSCs account only approximately 0.001% to 0.01% of the nucleated cells in the bone marrow [1]. Recently, it has been demonstrated that MSCs are also present in umbilical cord and placenta [7,8]. This profoundly increases the availability of MSCs, but *ex vivo* expansion remains an indispensable procedure to obtain sufficient amounts of MSCs for cell therapies and tissue engineering. 

MSCs have long been considered as expandable stem cells. However, recent studies indicate that MSCs age rapidly and undergo considerable changes in cell morphology and production of paracrine factors during culture expansion [9]. Oct4, Sox2 and Nanog are main transcription factors that govern embryonic stem cells self-renewal and pluripotency [10,11]. They are also expressed in MSCs and are involved in their multipotency [12]. Associated with morphological changes, rapid down-regulated expressions of these genes have been detected in MSCs during culture expansion [13]. Previous studies suggest that limited culture expansion of MSCs does not cause alterations in their genetic DNA sequences [14]. However, the epigenetic status of MSCs appears to be unstable in culture. Previous studies indicate that culture expansion of MSCs caused deacetylation of histone H3-K9 and 14 at promoters of pluripotent genes [13,22,23], which was associated with the appearance of aging signs [13]. Meanwhile, no evident changes in DNA methylation were found in the promoter regions of the pluripotent genes [13]. 

In this study, we attempted to use a histone deacetylase (HDAC) inhibitor trichostatin A (TSA) to suppress the reduction of histone acetylation in human MSCs (hMSCs) during culture expansion thus maintaining their primitive properties. We found that low concentrations of TSA significantly inhibited morphological changes of hMSCs that otherwise occurred during cell passaging. In addition, TSA-treated MSCs grew much faster. Moreover, TSA stabilized the expression of pluripotent genes and their histone H3 acetylation levels in lysine (K) 9 and K14 in the promoter regions. Our results suggest that TSA may be used as an effective agent to maintain the primitive feature of MSCs in culture expansion. 

## Materials and Methods

### Cell isolation and culture

Human MSCs were isolated from human placenta as described previously [13]. Briefly, term (38–40 weeks’ gestation) placentas from healthy donors were harvested with written informed consent and the procedure was approved by the Ethics Committee of Xili Hospital. The placental tissue was washed several times with cold phosphate-buffered saline (PBS) and then mechanically minced and enzymatically digested with 0.25% trypsin for 30 minutes at 37 °C in a water bath. The digest was subsequently pelleted by centrifugation and resuspended in a growth medium consisting of Dulbecco’s modified Eagle’s medium (DMEM, Gibco-Invitrogen) supplemented with 10% fetal bovine serum (FBS; Gibco-Invitrogen) and antibiotics. Cells were seeded on uncoated polystyrene dishes and incubated in the growth medium at 37 °C with 5% CO_2_. Medium was replaced every 2 days. When reaching 80% confluence, the cells were lifted by incubating with 0.25% trypsin/EDTA and sub-cultured. 

### TSA Treatment of hMSCs

To obtain optimal concentrations of TSA for hMSCs, TSA at concentrations of 0, 6.25 nM, 12.5 nM, 25 nM, 50 nM, 100 nM, 200 nM and 300 nM (dissolved in dimethyl sulfoxide, DMSO) was added to the growth medium. Equal volumes of DMSO alone were used as control. Human MSCs were cultured in 24-well plates at a concentration of 1x10^4^ cells per well in the presence of TSA or DMSO alone and incubated for 3 days. Then the cells were collected and counted with a hemacytometer. 6.25 nM TSA was chosen as an optimal concentration to stabilize histone acetylaton of hMSCs in culture for subsequent experiments. 

### Cell proliferation and cell cycle analysis

1x10^5^ cells per well of passage 1 hMSCs were seeded in six-well plastic tissue culture plates in triplet wells in the growth medium in the presence of 6.25 nM TSA or vehicle DMSO and incubated. Medium was changed every 2 days. When one of the wells reached 80% confluence, cells in all wells were harvested individually after trypsinization, counted with a hemacytometer and 1x10^5^ cells per well were re-seeded to a new six-well tissue culture plate. Cumulative cell numbers from passage 2 to passage 10 were calculated. Cells grown to full confluence (passages 6 and 10) and at the first day after passaging (passages 7 and 11 in ^~^30% confluence) were harvested for cell cycle analysis. Cells were fixed with 70% ethanol chilled at -20°C for 2 h, washed with PBS and re-suspended in a buffer containing 100 µg/mL propidium iodide and 10 µg/mL RNase A for at least 30 min in dark. Cells were then analyzed by flow cytometry (BD Biosciences).

### Western blotting

Western bloting was performed to assess the expression levels of cell cycle proteins using a method as previously described [15]. Briefly, cell lysates were prepared using a lysis buffer containing 1% Triton X-100 and proteinase inhibitors (Sigma-Aldrich). Equal amounts of total protein were separated on a 12% SDS-polyacrylamide gel and transferred to nitrocellulose membranes. Membranes were incubated overnight at 4°C with corresponding antibodies against cyclin D1, cyclin B1 and p21 (Santa Cruz), respectively. The bound antibodies were visualized using an ECL kit according to the manufacturer’s instructions. 

### MSC differentiation assays

Passage 1 hMSCs were grown in the presence of TSA (at 6.25 nM) or equal amount of vehicle DMSO to passage 6. Then the cells were incubated in adipogenic, osteogenic and chondrogenic induction media [13,15], respectively, for 3 weeks. The adipogenic induction medium contained 10^–6^ M dexamethasone, 10 µg/mL insulin and 100 µg/mL 3-isobutyl-L-methylxantine (Sigma). Cells were finally stained with Oil Red-O to detect lipid. The osteogenic medium contained 10^–7^ M dexamethasone, 50 µg/ml ascorbic acid and 10 mM β-glycerophosphate (Sigma). Cells were finally stained using Alzarin Red for calcium deposition. For chondrocyte differentiation, pellet hMSCs were cultured in DMEM (high glucose) containing 10^–7^ M dexamethasone, 50 µg/ml ascorbate-2-phosphate, 100 µg/ml pyruvate (Sigma), 10 ng/ml TGF-β1 (R&D Systems) and 50 mg/ml ITS Premix (BD Biosciences, 6.25 µg/ml insulin, 6.25 µg/ml transferrin, 6.25 ng/ml selenious acid, 1.25 mg/ml bovine serum albumin and 5.35 mg/ml linoleic acid). Medium was changed every 2 days for 3 weeks. The pellet was fixed, embedded and sectioned for H&E and toluidine blue (Sigma) staining, respectively. 

### RNA extraction and Real-Time PCR

Total RNA was extracted from hMSCs with TRIzol (Invitrogen) following the manufacturer’s instructions. First-strand cDNA was prepared by reverse transcription using Superscript II reverse transcriptase (Invitrogen) and oligo(dT) primers and stored at minus 20°C until use. Real-Time PCR was performed using SYBR Premix Ex Taq II (TaKaRa) on an ABI 7300 QPCR System for the expression of Oct4, Sox2, CD133, TERT, REX1, Nanog, alkaline phasphatase (ALP) and osteopontin (OPN) using primers previously described [13]. As an internal control, levels of glyceraldehyde-3- phosphate dehydrogenase (GAPDH) were quantified in parallel with target genes. Normalization and fold changes were calculated using the ΔΔCt method [15]. 

### Chromatin immunoprecipitation

Chromatin immunoprecipitation (ChIP) assay was performed using an Acetyl-Histone H3 Immunoprecipitation Assay Kit (Millipore) following the manufacturer’s protocol [13]. 1x10^6^ cells were used for each reaction. Histone acetylation was determined using specific antibodies against acetylated histone H3 at K9 and K14, respectively. After chromatin immunoprecipitation, DNA was extracted with a standard procedure (phenol/chloroform/isoamilic alcohol 25:24:1), and subsequently measured by quantitative fluorescent PCR analysis using SYBR Premix Ex Taq II (TaKaRa) on an ABI 7300 QPCR System. Primer targets were within 500 bp upstream of the gene transcription start site and primer sets were as follows: TERT forward 5’- GGCTCCCAGTGGATTCGC-3’, reverse 5’-GGAGGCGGAGCTGGAAGG- 3’; Sox2 forward 5’-AGTTGGACAGGGAGATGGC- 3’, reverse 5’-AACCTTCCTTGCTTCCACG-3’; Oct4 forward 5’-CTTCCACAGACACCATTGCC-3’, reverse 5’-AGTCCCACCCACTAGCCTTG- 3’. Data were analyzed using Percent Input Method (Invitrogen) following the instruction. 

## Results

### Low concentrations of TSA promotes proliferation and retains primitive features of hMSCs

**Figure 1:**
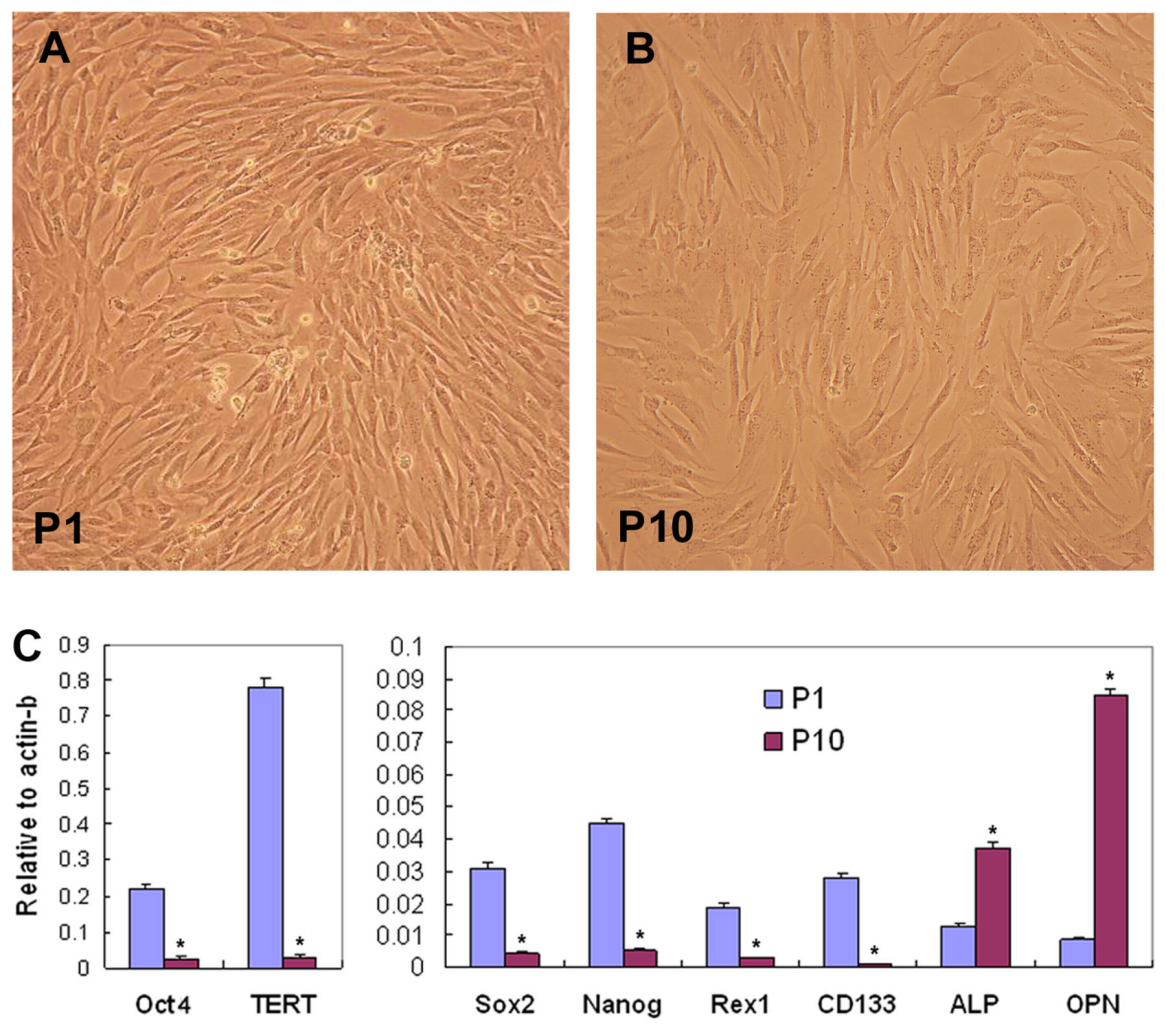
Changes of MSCs in culture. Cultured in growth medium, MSCs in passage (P) 10 were larger and fatter compared to cells in P1 (A & B). Real-Time PCR analysis showed expressional changes of genes involved in stem cell pluripotency and osteogenic differentiation (C). The experiment was repeated three time with similar results, and representative results from one experiment were shown (*P<0.01). ALP, asalkaline phosphatase; OPN, osteopontin.

With successive passages of hMSCs in plastic tissue culture dishes as monolayer, the shape of hMSCs became larger and fatter. In accordance with the morphological changes, Real-Time PCR analysis showed marked decreases of expression levels of pluripotent genes Oct4, Sox2, CD133, TERT, REX1 and Nanog, and increased levels of osteogenic genes ALP and OPN in passage 10 hMSCs compared to hMSCs in passage 1 (Figure 1). 

**Figure 2:**
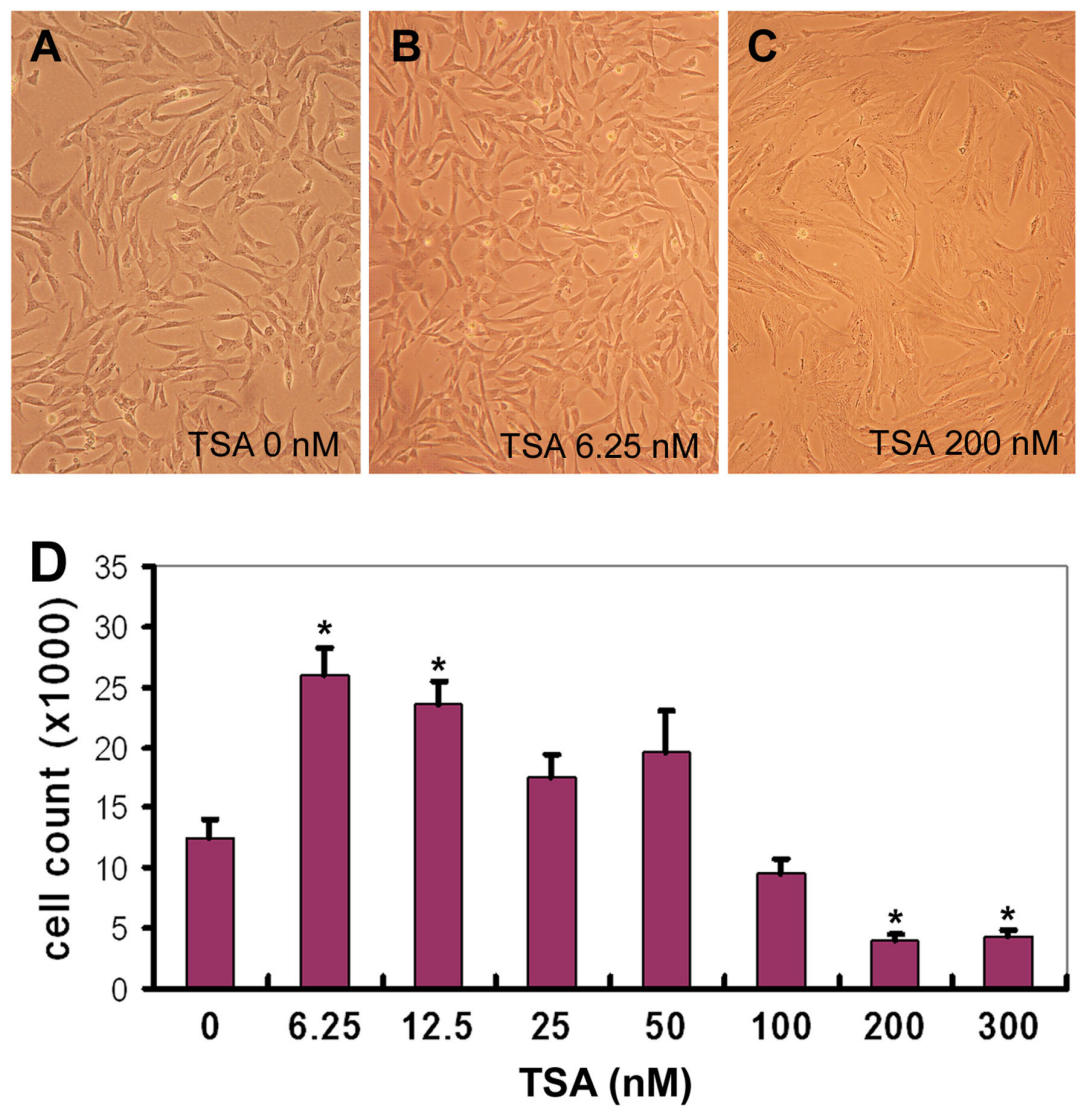
TSA treatment of hMSCs. Human MSCs were grown in the presence of different concentrations of TSA (0^~^300 nM) for 3 days and photographed under microscope. Reprehensive images were shown (A-C). Then the cells were detached and counted. Triplet wells were used for each experiment (D, n=4, * *P*<0.01).

We proposed that TSA could inhibit the decline of histone acetylation in pluripotent genes and thus retained the primitive properties of hMSCs. To test this, hMSCs were incubated in the growth medium supplemented with TSA at 0, 6.25, 12.5, 25, 50, 100, 200 and 300 nM for 3 days. We found that low concentrations of TSA (6.25 nM and 12.5 nM) increased the cell number by 2 folds (Figure 2B and D, *P*<0.01), and did not cause detectable changes in cell morphology; however, excessive amounts of TSA (200 or 300 nM) decreased hMSC proliferation and lead to significant changes in cell morphology, such as larger and flatter cell body in culture (Figure 2C and D, *P*<0.01). Similar results were obtained with hMSCs derived from three donors. 

### TSA Stabilizes Histone Acetylation and the Expression of Pluripotent Genes in hMSCs

**Figure 3:**
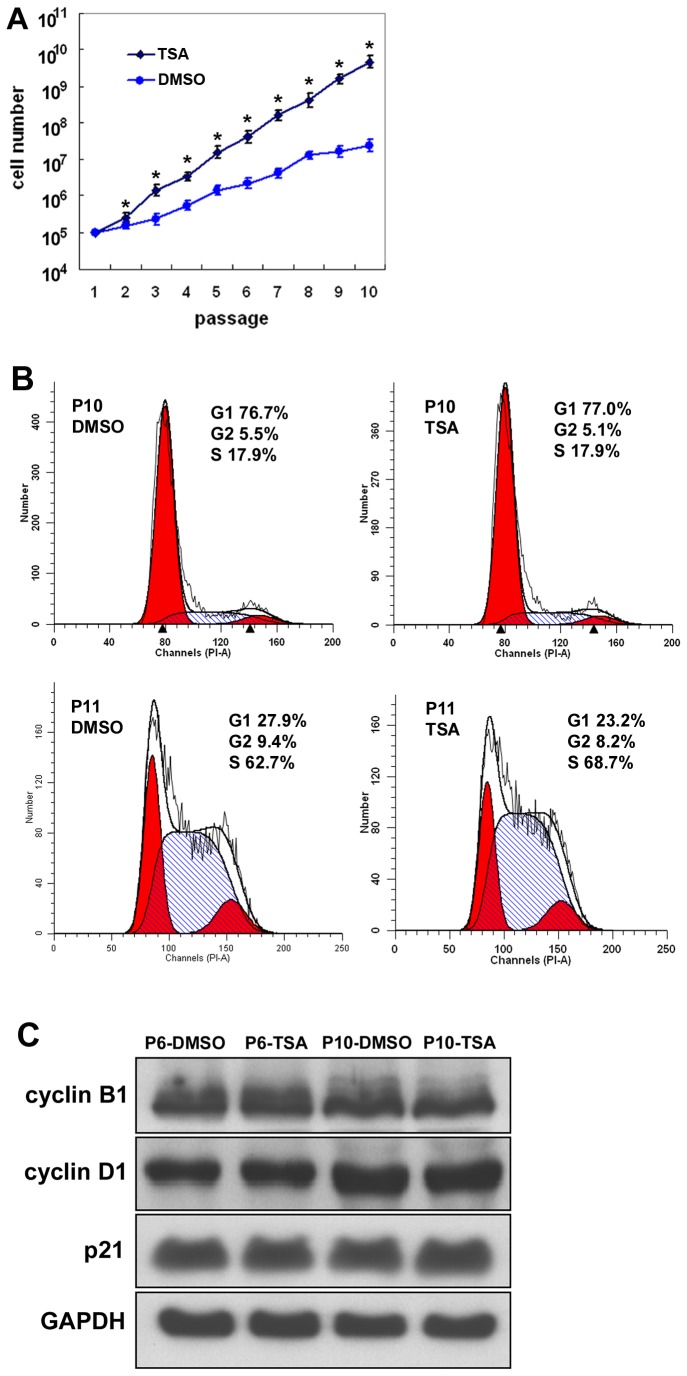
Influence of TSA on the growth and cell cycle of hMSCs. Passage (P)1 hMSCs were grown in the presence of TSA (at 6.25 nM) or vehicle DMSO to passage 10. (A) Cumulative cell numbers were counted (n=3, *P<0.01). (B) Cell cycle analysis of passage 10 hMSCs in full confluence showed similar percentages of hMSCs arrested in G1 phase (upper panel). When the cells were passaged to new culture plates (passage 11) and incubated in the growth medium for 24 hours (^~^30% confluence), a higher percentage of TSA-treated hMSCs were found in S phase (lower panel). (C) Western blot analysis of hMSCs in passage 6 and passage 10 hMSCs in full confluence for the expression of cell cycle proteins cyclin B1, cyclin D1 and p21. The experiments were repeated three times with similar results and representative results from one experiment were shown.

**Figure 4:**
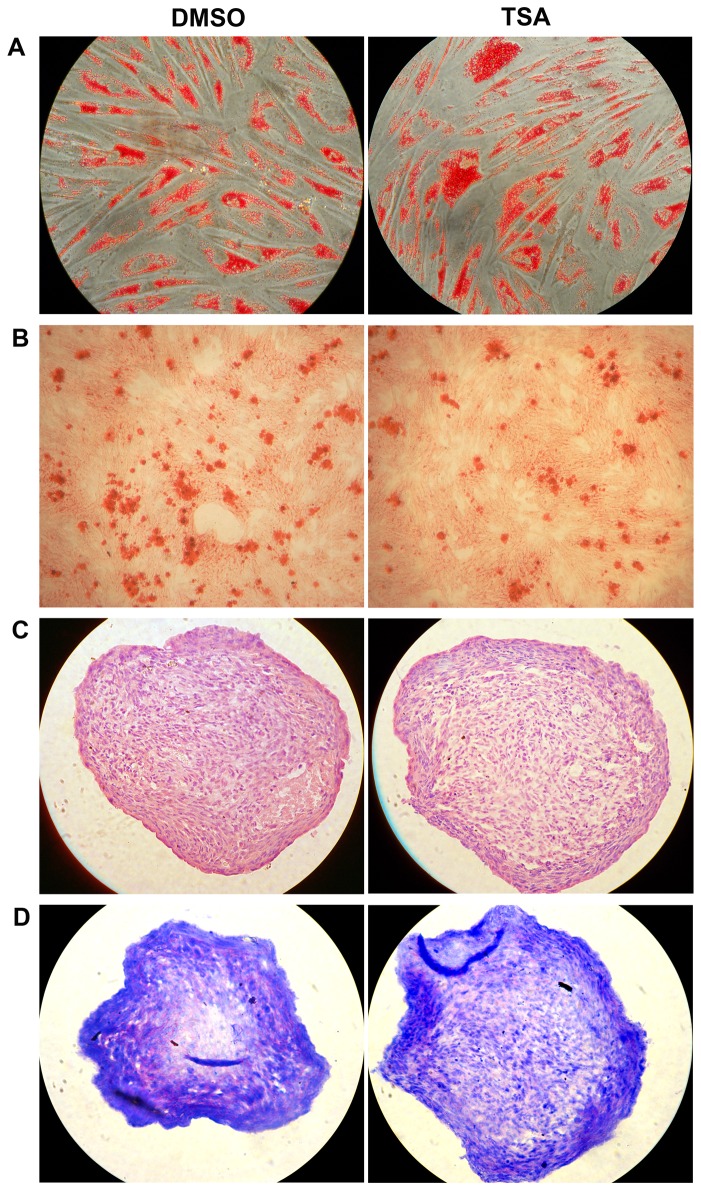
Differentiation of hMSCs. Passage (P)1 hMSCs were grown in the presence of TSA (at 6.25 nM) or vehicle DMSO to passage 6. Then the cells were incubated in adipogenic (A), osteogenic (B) and chondrogenic (C and D) induction media, respectively. MSCs differentiated into adipocytes (A, with oil red staining), osteoblasts (B, with Alizarin Red S staining) and chondrocytes (cultured in micromass and sectioned: C, with H&E staining; D, with toluidine blue staining showing pink extracellular matrix proteoglycans). The experiments were repeated three times with similar results and representative results from one experiment were shown.

We then analyzed the long term influences of TSA on hMSCs. Human MSCs were cultured in the presence of TSA (at 6.25 nM) or an equal amount of DMSO (the dissolvent of TSA) in the growth medium from passage 1 to passage 10. Progressive changes in cell morphology with successive cell passages as described earlier were observed in hMSCs treated with DMSO alone. However, the morphological changes did not occurred in hMSCs cultured in the presence of TSA. Meanwhile, there was a profound increase in the cumulative cell number of hMSCs in culture in the presence of TSA (Figure 3A, P<0.01). To investigate whether transformation occurred in TSA-treated cells, we examined cell contact inhibition in cell growth in hMSCs after successive TSA treatment. Similar to DMSO-treated hMSCs, TSA-treated hMSCs stopped proliferating when they reached full confluence and no multi-layer foci were found in the culture. Cell cycle analysis of passage 10 hMSCs treated with DMSO or TSA showed similar percentages of cells arrested in G1 phase (76% versus 77%, n=3, *P*>0.05, Figure 3B) when they reached full confluence. However, when cells were passaged to new culture plates (passage 11) and incubated in the growth medium for 24 hours (in ^~^30% confluence), a higher percentage of TSA-treated hMSCs entered S phase compared to DMSO-treated cells (67% versus 61%, n=3, *P*<0.05, Figure 3B). We further examined the expression levels of cell cycle proteins in passage 6 and passage 10 hMSCs in full confluence by Western blot, and the results showed similar amounts of cyclin D1, cyclin B1 and p21 in DMSO- and TSA-treated cells (Figure 3C). We also examined the multipotent differentiation potential of hMSCs into adipocytes, osteoblasts and chondrocytes, which has been considered as a typical feature of MSCs, and found that similar differentiations into these three cell lineages occurred in TSA-treated hMSCs, compared to DMSO-treated hMSCs (Figure 4A-D). 

**Figure 5:**
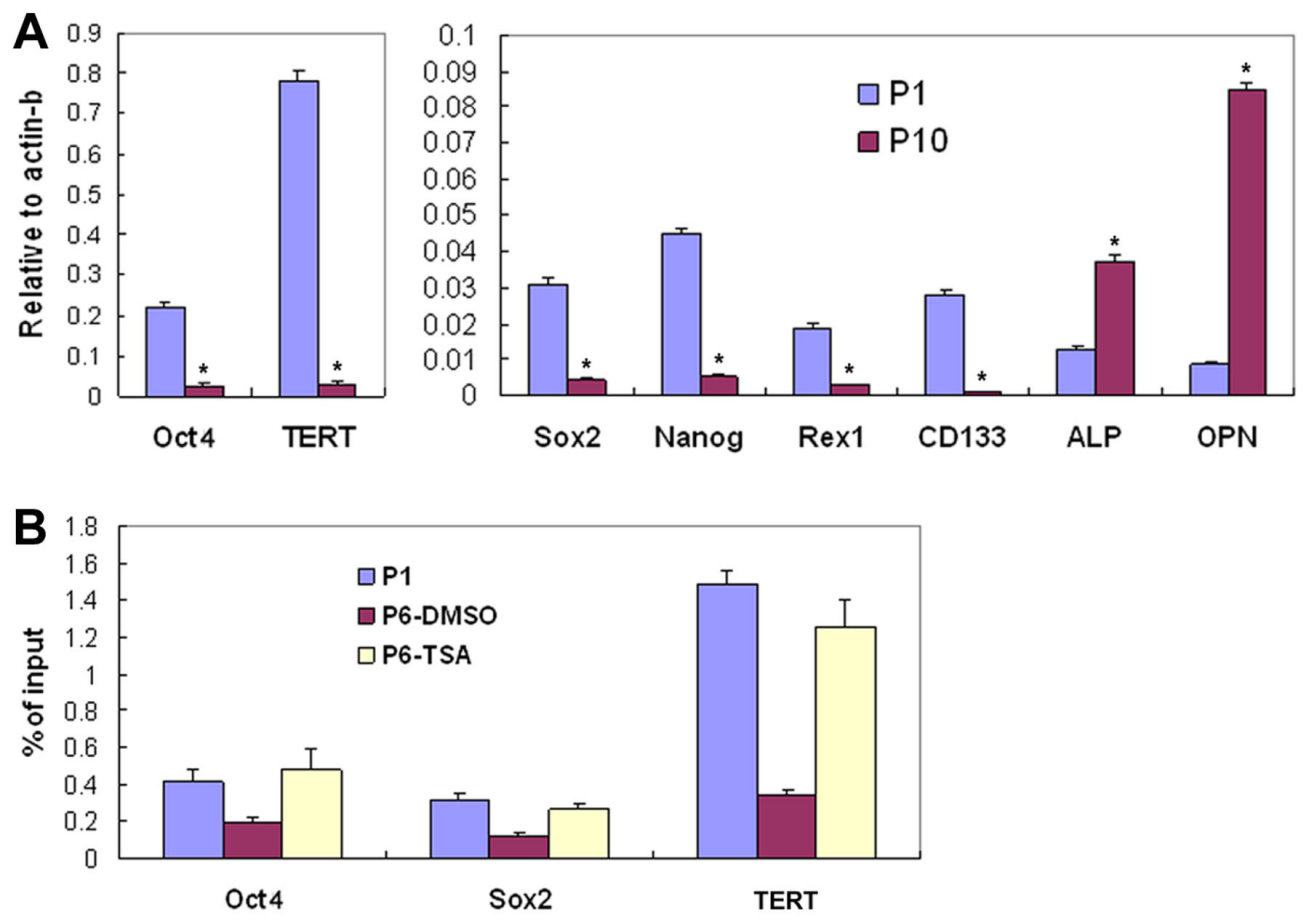
Influence of TSA on the expression and histone H3 acetylation of pluripotent gene in hMSCs. Passage (P)1 hMSCs were grown in the presence of TSA (at 6.25 nM) or vehicle DMSO to passage 6. (A) Real-Time PCR analysis of Oct4, TERT, Sox2, Nanog, REX1 and CD133 genes of the cells in P1 and P6 (A, n=3, *P<0.01 when P6-DMSO compared to P1 and to P6-TSA). (B) Acetylation of histone H3 in K9 and K14 in Oct4, Sox2 and TERT genes in DMSO- or TSA-treated hMSCs (*P<0.01 when P6-DMSO compared to P1 and to P6-TSA). The experiments were repeated three times with similar results.

Next, we examined the expression of pluripotent genes in hMSCs in the above cultures. We found that TSA significantly inhibited the down-expression of Oct4, TERT, Sox2, Nanog, REX1 and CD133 genes from passage 1 to passage 6 hMSCs, which occurred in hMSCs treated with vehicle DMSO alone (Figure 5A, P<0.01). Finally, we examined histone H3 acetylation in K 9 and K14 in the promoter regions of TERT, Sox2 and Oct4 genes in hMSCs. Compared to hMSCs in passage 1, hMSCs cultured in the presence of DMSO alone in passage 6 showed significantly decreased histone H3 acetylation levels of the pluripotent genes in K 9 and K14 (Figure 5B, P<0.01). In the presence of TSA (at 6.25 nM), the acetylation levels of histone H3 in K 9 and K14 of these genes in passage 6 hMSCs showed no significant decreases (Figure 5B, P>0.05). 

## Discussion

To achieve maximum therapeutic effects of MSCs in tissue repair/regeneration, it is a pre-requirement to retain their primitive properties during *ex vivo* expansion. However, several previous studies have indicated that MSCs age quickly and gradually lose their multipotent differentiation potential in culture [13,16]. Therefore, it is a crucial issue to develop optimal culture conditions to maintain the quality of MSCs for clinical uses. 

Previous studies indicate that the acetylation of K9 and K14 in histone H3 is crucial for gene transcription. It is required for the recruitment of transcription factor II D (TFIID), one of general transcription factors that bind the TATA box in the core promoter to initiate gene transcription [17,18]. In our previous study, we found that decreases of acetylation levels in K9 and K14 of histone H3 were closely associated with the aging and spontaneous differentiation of hMSCs [13]. 

In this study, we showed that TSA at low concentrations was potent in maintaining the histone acetylation states of K9 and K14 in histone H3 of pluripotent genes in hMSCs, thus stabilizing the expression of pluripotent genes and retaining the primitive features of the cells during culture expansion. Moreover, hMSCs cultured in the presence of low concentrations of TSA grew faster with consistent cell morphology. TSA, which was initially used as an antifungal antibiotic, has recently been found to be a potent and specific inhibitor of HDAC activity [19,20]. It selectively inhibits the class I and II, but not class III, mammalian HDAC families of enzymes [21]. Previous studies suggest that TSA modulates a wide variety of cellular activities such as cell differentiation and proliferation depending on cell types and their functional states [20]. TSA at concentrations of 200^~^300 nM has been found to exhibit pronounced suppressive effect on breast cancer cells with immeasurable toxicities [19]. In this study, however, TSA at concentrations of 200^~^300 nM caused evident morphological changes in addition to growth inhibition. It appears that MSCs are very sensitive to TSA. 

Previous studies suggest that histone acetylation is critically involved with *ex vivo* aging of MSCs. Culture expansion of MSCs caused deacetylation of histone H3-K9 and 14 in promoters of pluripotent genes [13,22,23]. Meanwhile, no significant changes were found in levels of DNA methylation in promoters of the genes [13]. In this study, preventive application of low doses of TSA markedly prevented the deacetylation of histone H3-K9 and 14 and the appearance of aging signs. These results suggest that low dose TSA may serve as an effective supplement to hMSC culture to stabilize their histone acetylation, and thus keep their primitive features. 
